# Efficacy of Terbinafine and Itraconazole Combination Therapy Versus Terbinafine or Itraconazole Monotherapy in the Management of Fungal Diseases: A Systematic Review and Meta-Analysis

**DOI:** 10.7759/cureus.48819

**Published:** 2023-11-14

**Authors:** Syed Hassan Tanvir Ramzi, Syed Abdullah Arif, Abdul Majid, Satesh Kumar, Hooria Shumail, Faiza Qudsia, Yumna Zainab, Giustino Varrassi, Mahima Khatri

**Affiliations:** 1 Medicine, Bakhtawar Amin Memorial Hospital, Multan, PAK; 2 Medicine, Allama Iqbal Medical College, Lahore, PAK; 3 Internal Medicine, Khyber Medical College, Peshawar, PAK; 4 Medicine and Surgery, Shaheed Mohtarma Benazir Bhutto Medical University, Karachi, PAK; 5 Medicine, King Edward Medical University (KEMU), Lahore, PAK; 6 Medicine, Multan Medical and Dental College, Multan, PAK; 7 Pain Medicine, Paolo Procacci Foundation, Rome, ITA; 8 Medicine and Surgery, Dow University of Health Sciences, Karachi, PAK

**Keywords:** monotherapy, meta-analysis, fungi, fungal, itraconazole, terbinafine

## Abstract

Fungal infections constitute a common dermatological illness rampant in underdeveloped countries. Combination drug therapy is becoming increasingly well-established owing to drug resistance because of monotherapy. Different studies have been conducted previously to compare the medical regimens for the treatment of fungal infections. However, there is insufficient research on the difference in cure rates and recurrence rates with each regimen. To the best of our knowledge, this meta-analysis is the first to compare the effect of the most widely used oral antifungal medications and their combination usage. A meta-analysis of randomized controlled trials (RCTs) assesses the efficacy of terbinafine or itraconazole monotherapy versus combination therapy in fungal diseases. We queried PubMed and Cochrane Central from their inception to April 2022 for published studies, RCTs, and observational studies without any language restriction that compared itraconazole and terbinafine combination therapy with monotherapy in patients with fungal infections. The results from the studies were presented as risk ratios (RRs) with 95% confidence intervals (CIs) and were pooled using a random-effects model, and a p-value of ≤0.05 was considered significant for the analysis. Endpoints of interest included cure rates and recurrence rates. Cure rates were increased significantly for combination therapy compared to terbinafine monotherapy (RR=2.01 (1.37, 2.94); p=0.0003; I2=67%). On sensitivity analysis, a significant association was observed between combination therapy and itraconazole monotherapy in terms of cure rates (RR=1.91 (1.41, 2.57); p<0.0001; I2=0%) and recurrence rates (RR=0.08 (0.02, 0.44); p=0.003; I2=0%). The findings of this meta-analysis suggest that itraconazole and terbinafine combination therapy has a better cure rate when compared to terbinafine monotherapy.

## Introduction and background

In recent years, fungal infections have increased with an estimated global prevalence of 20-25%, thereby causing significant global mortality and posing therapeutic challenges [1]. These skin, body, and nail infections are caused by various fungi and are classified according to their location on the body, with dermatophytes being the most common cause. Common dermatological conditions include tinea capitis, tinea pedis, and onychomycosis. Oral or topical antifungal medications can effectively treat these conditions according to the severity of symptoms [2]. Medications such as terbinafine, itraconazole, fluconazole, and luliconazole are currently under investigation to treat fungal diseases in clinical trials. Terbinafine, an allylamine antifungal drug, is considered the first-line treatment for tinea caused by trichophyton infections because of its fungicidal properties [3]. Itraconazole is also recommended for pulse therapy to treat fungal diseases owing to its extensive protein-binding properties, which ensures that its concentration at the site of infection remains higher than the corresponding plasma concentration [4].

Resistance to these drugs becomes increasingly prevalent when standard doses and durations are used. This is attributable to the decreased effective concentration of terbinafine and itraconazole because of their extensive accumulation in the skin and adipose tissue [4,5]. Thus, widespread resistance to conventional antifungals, combined with increasing clinical failure rates, necessitates the development of an effective combination of antifungal agents capable of achieving a rapid clinical and mycological cure. As a result, combination treatment is a well-established concept for improving therapeutic efficacy and overcoming drug resistance by combining two or more medications' synergistic and additive effects. In vitro, terbinafine and itraconazole have been shown to have a synergistic effect against a wide range of dermatophytes and non-dermatophyte fungi [6].

In this meta-analysis, we report findings after analyzing the whole available literature on this topic. Given the rise in antifungal resistance, our meta-analysis compares the clinical effectiveness of terbinafine and itraconazole combination therapy with monotherapy (terbinafine or itraconazole). Based on an extensive and thorough literature search, we conclude that this meta-analysis is the first of its kind investigating the therapeutic effects of combination therapy (terbinafine + itraconazole) with terbinafine or itraconazole monotherapy in fungal diseases. The primary objective is to provide experimental evidence for a clinical application of terbinafine + itraconazole combination treatment for patients with fungal disease.

## Review

Methods

This meta-analysis was performed under the Preferred Reporting Items for Systematic Review and Meta-Analyses (PRISMA) guidelines [7].

Data Sources, Strategy, and Study Selection 

**Figure 1:**
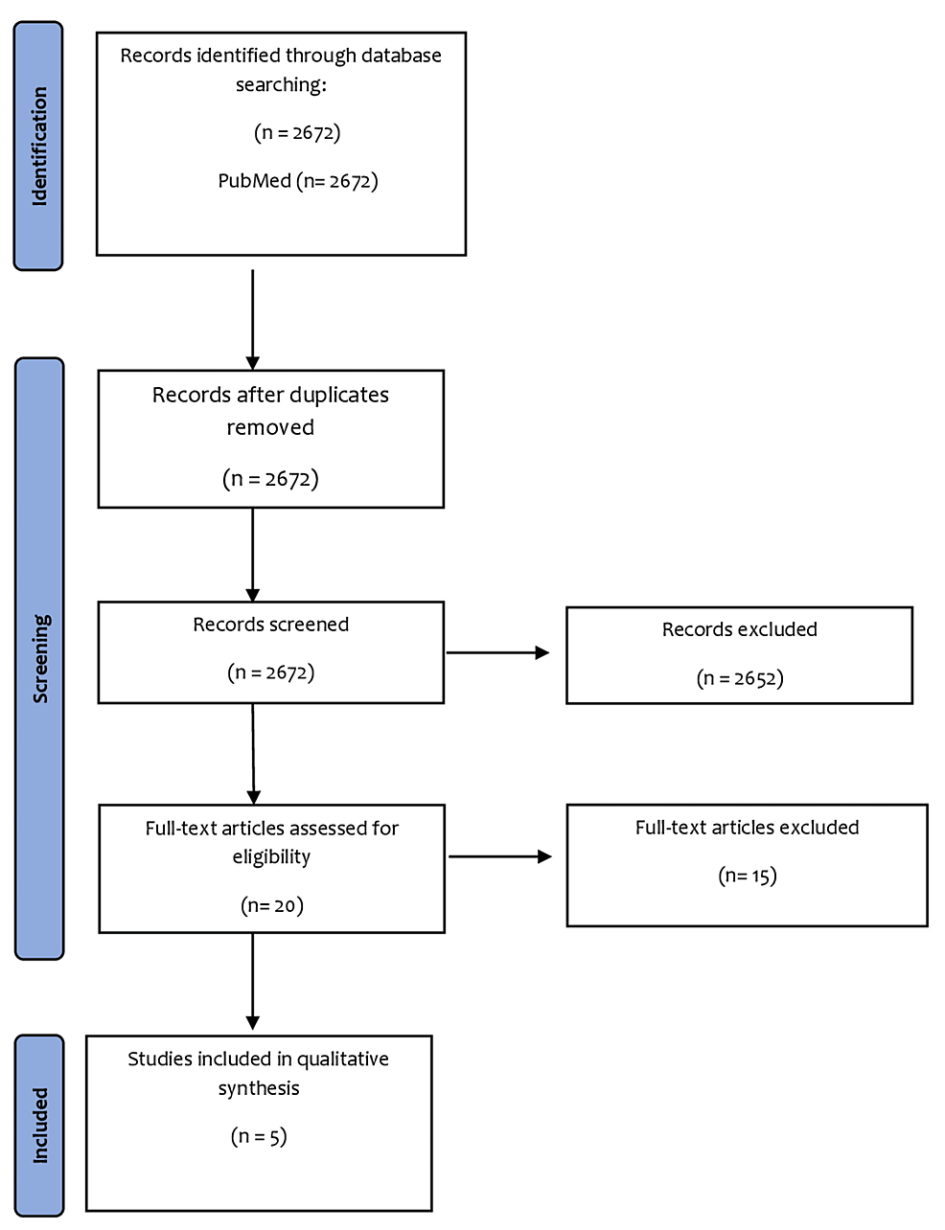
PRISMA flow diagram PRISMA: Preferred Reporting Items for Systematic Reviews and Meta-Analyses

Two independent reviewers (SHTR and SAA) performed an electronic search of PubMed and Cochrane CENTRAL from their inception to April 2022 using a comprehensive search strategy that involved generic, pharmaceutical, and trade names and abbreviations of the drugs, along with MeSH terms and Boolean operators ‘AND’ and ‘OR.’ Any dispute between the two independent reviewers (SHTR and SAA) regarding study selection was resolved by discussion and a mutual consensus with a third investigator (SK). Grey and white literature were also searched. Bibliographies of relevant review articles were also queried. The predefined eligibility criteria for our meta-analysis were: (a) published and randomized controlled trials (RCTs) or observational studies; (b) compared terbinafine or itraconazole alone vs. terbinafine and itraconazole therapy; and (c) reported at least one of the following outcomes of cure rate and recurrence rate. A total of 2,672 studies were thoroughly screened, after which five articles were selected for analysis as shown in the PRISMA flowchart (Figure 1).

Data Extraction and Quality Assessment of Studies

The studies yielded by our search strategy were cross-verified by the two independent reviewers (PK and HT) and compiled in Endnote Reference Library (version X7.5; Clarivate Analytics, Philadelphia, PA) software, where duplicates were searched and removed. All full texts of the remaining articles were then thoroughly reviewed to extract the outcomes and their raw data. In addition, the reference sections of these full-text articles were manually screened for any relevant studies that might have been missed during the electronic search. In cases where raw data was available, the summary events were proportionated to calculate RRs with 95% confidence intervals (CIs). Moreover, study and patient baseline characteristics were extracted and reported in Table 1 of the text and Table 2, respectively. To assess the quality of studies across six domains [selection bias, performance bias, detection bias, attrition bias, reporting bias, and other bias], we used Cochrane Collaboration’s risk of bias tool for RCTs as shown in Table 3 [8]. In addition, heterogeneity in effect sizes was assessed using Higgin’s I2 statistics, where the I2 value of greater than 50 % was considered significant [9]

**Table 1:** Characteristics of the included studies NA: Not available

Study	Year	No. of Patients	Inclusion Criteria	Control	Intervention	Follow-up
Singh et al. [9]	2020	275	KOH positive cases of tinea cruris, corporis and faciei	NA	Terbinafine 250 mg/day, Itraconazole 200 mg/day in two divided doses, Terbinafine 250 mg + Itraconazole 200 mg, Terbinafine 500 mg/day in two divided doses, Itraconazole 400 mg/day in two divided doses	8 weeks
Zhang et al. [10]	2021	178	Age 15-50 years, Clinically diagnosed, in vitro fungal culture positive, microscopic examination positive, informed consent given	Terbinafine or Itraconazole monotherapy	Terbinafine and Itraconazole combination therapy	4 weeks
Gupta et al. [11]	2013	106	Mycologically cured at 48 weeks after the beginning of therapy based on a last observation carried forward (LOCF) analysis, both clinically and mycologically assessed after week 48	Terbinafine (continuous or intermittent) or Itraconazole (pulsed) monotherapy	Terbinafine and Itraconazole combination therapy	7 years
Gupta et al. [12]	2001	190	Age 18 years, clinical diagnosis of onychomycosis, the etiological organism had to be a dermatophyte	3 or 4 pulses of terbinafine	Sequential 2 pulses of Itraconazole followed by 1 or 2 pulses of terbinafine	72 weeks
Sharma et al. [13]	2019	60	Clinically diagnosed and KOH-positive (septate hyphae) patients of tinea corporis, tinea cruris, or tinea faciei	Terbinafine or itraconazole monotherapy	Terbinafine and Itraconazole combination therapy	9 weeks

**Table 2:** Baseline characteristics of the included studies SD: Standard deviation, NA: Not available

Author, Year	Therapy Type and Doses	Age in Years (Mean ± SD)	Males (%)	Duration of Disease	Ethnicity	Causative Organisms/Type of Disease
Singh et al. (2020) [9]	Terbinafine 250 mg/day	32.45±11.41	70.2	NA	Indian	Tinea corporis, Tinea cruris, Tinea faciei,
	Itraconazole 200 mg/day in two divided doses	28.06±12.54	72.3			
	Terbinafine 250 mg + Itraconazole 200 mg	29.67±10.12	75			
	Terbinafine 500 mg/day in two divided doses	27.02±10.68	68.8			
	Itraconazole 400 mg/day in two divided doses	29.65±11.73	84.8			
Zhang et al. (2021) [10]	Terbinafine	32.35 ± 9.89	41	2.10 ± 0.86 Years	Chinese	Tinea manuum, Tinea pedis, Tinea corporis, Tinea cruris Onychomycosis, Sporotrichosis, and Fungal genital inflammation
	Itraconazole	33.07 ± 10.68	26	2.24 ± 1.09 Years		
	Terbinafine +Itraconazole	32.61 ± 10.83	46	2.28 ± 1.13 Years		
Gupta et al. (2013) [11]	Terbinafine, Itraconazole, Terbinafine +Itraconazole	55 ± 15	65	NA	Canadian	Trichophyton rubrum, Trichophyton mentagrophytes
Gupta et al. (2001) [12]	Sequential pulse therapy with 2 pulses of itraconazole followed by 1 or 2 pulses of terbinafine	53.1 ± 0.18	44	5.7 ± 0.7 Years	Canadian	Trichophyton rubrum, Trichophyton mentagrophytes
	3 or 4 pulses of terbinafine	56.5 ± 0.13	66.6	8.0 ± 1.0 Years		
Sharma et al. (2019) [13]	Terbinafine, Itraconazole, Terbinafine +Itraconazole	14-20 Years = 8, 21-40 Years = 34, 41-60 Years = 18	58.3	13.06 Weeks	Indian	Trichophyton mentagrophytes, Trichophyton rubrum, Trichophyton Terrestre, Trichophyton verrucosum, Microsporum gypseum, Candida tropicalis, Aspergillus species,

**Table 3:** Quality assessment of the included studies

Quality variables	Gupta et al. (2001) [12]	Gupta et al.(2013) [11]	Sharma et al. (2019) [13]	Singh et al. (2020) [9]	Zhang et al. (2021) [10]
Random sequence generation	Low risk	Low risk	Low risk	Low risk	Low risk
Allocation concealment	Low risk	Low risk	Low risk	Low risk	Low risk
Blinding of participants and personnel	Low risk	Low risk	Low risk	High risk	Low risk
Blinding of outcome assessment	Low risk	Low risk	Low risk	High risk	Low risk
Incomplete outcome data	Low risk	Low risk	Low risk	Low risk	Low risk
Selective reporting	Low risk	Low risk	Low risk	Low risk	Low risk
Other bias	Low risk	Unclear risk	Low risk	Unclear risk	Unclear risk

Statistical Analysis 

All statistical analyses were conducted using RevMan (version 5.3; Nordic Cochrane Centre, Odense, Denmark). The forest plots of relevant outcomes were constructed to visually represent the RRs with 95% CIs and were pooled using the random-effects model.

Results

Out of 2,672 studies that were reviewed for eligibility, we selected five RCTs. Sharma et al. [13] enlisted 60 patients, who were provided with a combination of terbinafine and itraconazole and followed for nine weeks. Zang et al. included 178 patients who received a similar intervention but were monitored for four weeks. Gupta et al. [11] included 106 patients who received combined therapy for seven years, while the control group received terbinafine (continuous or intermittent) or itraconazole (pulsed) monotherapy. Gupta et al. [12] enrolled 119 patients who received Sequential 2 pulses of itraconazole followed by one or two pulses of terbinafine for 72 weeks, whereas Singh et al. [9] enrolled 275 patients who received terbinafine in two divided doses in addition to itraconazole for eight weeks with no control group.

Cure Rate in Combination Therapy Versus Terbinafine Monotherapy

**Figure 2:**
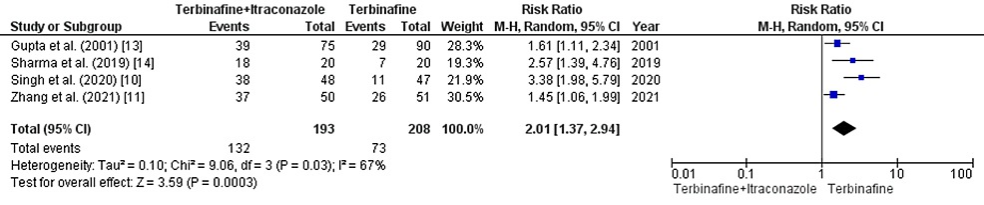
Cure rate in combination therapy versus terbinafine monotherapy Source: References [10,11,13,14]

In our pooled analysis, four studies (n=401) provided data for the outcome of the cure rate in terbinafine and itraconazole combination therapy versus terbinafine monotherapy. A pooled analysis of these studies revealed that terbinafine and itraconazole combination therapy is significantly associated with a higher cure rate than terbinafine monotherapy (RR=2.01 [1.37, 2.94]; p=0.0003; I2=67%) (Figure 2).

Cure Rate in Combination Therapy Versus Itraconazole Monotherapy

**Figure 3:**
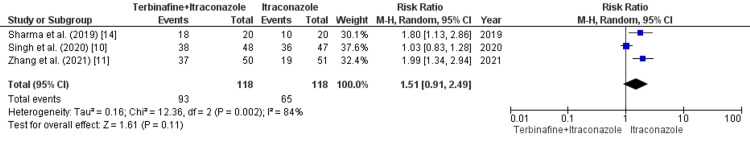
Cure rate in combination therapy versus itraconazole monotherapy Source: References [10,11,14]

In our pooled analysis, the cure rate in terbinafine and itraconazole combination therapy versus itraconazole monotherapy was reported in three studies (n=236). A meta-analysis of these studies revealed a statistically nonsignificant association between cure rate and terbinafine and itraconazole combination therapy (RR=1.51 (0.91, 2.49); p=0.11; I2=84%) (Figure 3).

Recurrence in Combination Therapy Versus Terbinafine Monotherapy

**Figure 4:**
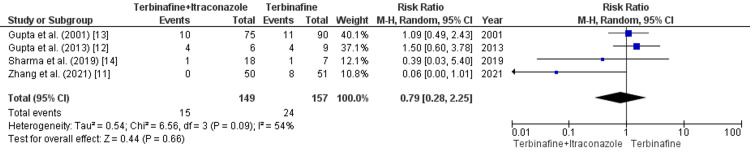
Recurrence in combination therapy versus terbinafine monotherapy Source: References [11-14]

In our pooled analysis, four studies (n=306) provided data for the recurrence rate outcome in terbinafine and itraconazole combination therapy versus terbinafine monotherapy. A meta-analysis of these studies revealed a statistically nonsignificant association between recurrence and terbinafine monotherapy (RR=0.79 [0.28, 2,25]; p=0.66; I2=54%) (Figure 4).

Recurrence in Combination Therapy Versus Itraconazole Monotherapy

**Figure 5:**
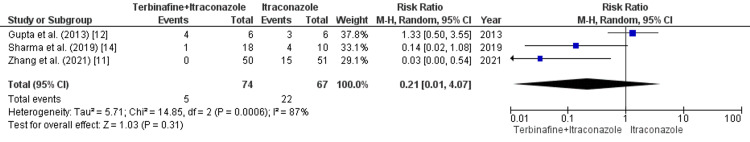
Recurrence in combination therapy versus itraconazole monotherapy Source: References [11,12,14]

In our pooled analysis, three studies (n=141) provided data for the outcome of recurrence in terbinafine and itraconazole combination therapy versus itraconazole monotherapy. A meta-analysis of these studies revealed a statistically nonsignificant association between recurrence and itraconazole monotherapy (RR=0.21 [0.01, 4.07]; p=0.31; I2=87%) (Figure 5).

Sensitivity Analysis

The cure rate in combination therapy versus terbinafine monotherapy: As a result of the significant heterogeneity, a sensitivity analysis was performed, and heterogeneity for terbinafine and itraconazole combination therapy was reduced from (I2=67%) to (I2=25%). A statistically significant association was revealed (p=0.0002). Singh et al. [10] primarily contributed to the increased heterogeneity.

The cure rate in combination therapy versus itraconazole monotherapy: As a result of the significant heterogeneity, a sensitivity analysis was performed, and heterogeneity for terbinafine and itraconazole combination therapy was reduced from (I2=84%) to (I2=0%). A statistically significant association was revealed (p<0.0001). Singh et al. [10] primarily contributed to the increased heterogeneity.

Recurrence in combination therapy versus terbinafine monotherapy: As a result of the significant heterogeneity, a sensitivity analysis was performed, and heterogeneity for terbinafine monotherapy was reduced from (I2=54%) to (I2=0%). Zhang et al. [11] primarily contributed to the increased heterogeneity.

Recurrence in combination therapy versus itraconazole monotherapy: As a result of the significant heterogeneity, a sensitivity analysis was performed, and heterogeneity for itraconazole monotherapy was reduced from (I2=87%) to (I2=0%). A statistically significant association was revealed (p=0.003). Gupta et al. [12] primarily contributed to the increased heterogeneity.

Discussion

This meta-analysis of five clinical trials [9-13] comprising more than 750 patients sought to compare the efficacy of terbinafine and itraconazole combination therapy with their monotherapies in managing fungal diseases. The outcomes observed were complete clinical and mycologic cure rates and recurrences. Cure rates with combination therapy were significantly higher than terbinafine monotherapy. Combination therapy was nonsignificantly associated with the cure rate of itraconazole and the recurrence rate of either drug. During sensitivity analysis, the removal of Singh et al. [9] from cure rate plots and that of Zhang and Gupta et al. [10,11] from recurrence rate plots revealed statistically significant associations and reduced heterogeneity, which likely arose because of the use of topical drugs, small study groups, lack of blinding, younger average ages, and variable follow-up durations in these studies.

Different studies have been conducted previously to compare the medical regimens for the treatment of fungal infections [5,15,16]; however, these only compare antifungal monotherapies, and there is insufficient research on the difference in cure rates and recurrence rates with each regimen. A previous systematic review compared the in-vitro effects of several antifungal combinations in dermatophytosis [17]; however, to the best of our knowledge, this meta-analysis is the first to compare the effects of itraconazole and terbinafine - two of the most commonly used antifungal medications [14] - with their combined usage, a comparison only studied in-vitro [18]. Three of the five clinical trials we analyzed reported both outcomes of cure rates and recurrences, while the remaining two studied one outcome each. Results from cure rates analysis were consistent with three of the included trials [11,13,14], while those of recurrence rates were similar to the results of two of these trials [11,13].

A combination of itraconazole and terbinafine can enhance clinical outcomes owing to their mechanisms by which they sequentially target ergosterol in fungal cell membranes during its formation pathway at two distinct steps. Synergism from their combination has also shown promising results in a pharmacokinetic model [19]. Emerging resistance against these drugs in pathogenic fungi [1,20,21] and their variable bioavailability in the human body [22,23] can limit the relative efficacy of their monotherapy, but factors other than resistance may have a role in treatment failures as well, like host-fungal interactions and virulence of the fungi [24,25]. A recent paper [26] suggested reserving combination therapy, despite being well-tolerated, as second-line treatment for onychomycosis, considering costs, conflicting results of included trials, and safety concerns, which may include drug interactions because of long-term azole antifungal usage [22,27].

During the sensitivity analysis for complete cure rate, the removal of Singh et al. [9] - which showed conflicting results for monotherapy versus combination therapy - reduced heterogeneity that likely occurred in response to topical antifungal application by some participants during the follow-up phase, thereby facilitating the effects of the oral drugs, the lack of blinding, or lower mean age compared to other studies. The heterogeneity in the outcome plot for recurrence in itraconazole versus combination treatment was minimized by omitting the Gupta et al. [11] trial from 2013, wherein the study groups were much smaller. Finally, removing Zhang et al. [10] from the outcome plot for recurrence in terbinafine versus combination therapy reduced heterogeneity, which may be attributable to the external application of powdered drugs during the study.

Several limitations may influence the results of this meta-analysis. First, the number of included studies and participants was considered insufficient. Second, participants from different geographic populations were included and may have responded differently to drugs. Third, doses of administered drugs were fixed instead of being calculated according to body mass. Fourth, multiple infective organisms with differing virulence were considered - although most were dermatophytes. Fifth, laboratory criteria for determining mycologic cure rates and criteria to determine clinical cure rates were contrasting across studies. Finally, different levels of blinding were applied in trials, and different brands of drugs were administered which may have differing efficacies.

Many cases of resistant superficial fungal infections are continuing to rise globally [21,28], and climate changes are occurring that particularly favor the growth of these organisms [29]. It is, therefore, essential to establish a well-tolerated treatment regimen that offers optimal therapeutic efficacy with minimal adverse effects, which may involve combining two or more drugs based on their synergistically enhanced outcomes. As our meta-analysis has shown statistically significant improved outcomes with a combination of itraconazole and terbinafine, it provides a reliable and effective option for the treating physicians.

## Conclusions

Our meta-analysis suggests that terbinafine and itraconazole combination therapy has statistically and significantly better efficacy in curing superficial fungal infections compared to terbinafine alone. Additional research in the future is required to examine and document side effects and toxicity levels in diverse populations to augment the findings of this analysis because of a limited number of studies and participants.
